# Structural and Functional Analysis of the ApolipoproteinA-I A164S Variant

**DOI:** 10.1371/journal.pone.0143915

**Published:** 2015-11-25

**Authors:** Jonathan Dalla-Riva, Jens O. Lagerstedt, Jitka Petrlova

**Affiliations:** Department of Experimental Medical Science, Lund University, Lund, Sweden; University of Padova, ITALY

## Abstract

Apolipoprotein A-I (apoA-I) is the main protein involved in the formation of high-density lipoprotein (HDL), it is the principal mediator of the reverse cholesterol transfer (RCT) pathway and provides cardio-protection. In addition to functional wild-type apoA-I, several variants have been shown to associate with hereditary amyloidosis. In this study we have performed biophysical and biochemical analyses of the structure and functional properties of the A164S variant of apoA-I (1:500 in the Danish general population), which is the first known mutation of apoA-I that leads to an increased risk of ischaemic heart disease (IHD), myocardial infarction and mortality without associated low HDL cholesterol levels. Despite the fact that epidemiologically IHD is associated with low plasma levels of HDL, the A164S mutation is linked to normal plasma levels of lipids, HDL and apoA-I, suggesting impaired functionality of this variant. Using biophysical techniques (e.g., circular dichroism spectroscopy and electron microscopy) to determine secondary structure, stability and pro-amyloidogenic property of the lipid free A164S apoA-I variant, our observations suggest similarity in structural properties between apoA-I WT and apoA-I A164S. However, the A164S apoA-I variant exhibits lower binding affinity to lipids but forms similar sized HDL particles to those produced by WT.

## Introduction

Apolipoprotein A-I (apoA-I) is the main protein involved in the formation of high-density lipoprotein (HDL), which is the principal mediator of the reverse cholesterol transfer (RCT) pathway and provides cardio-protection [1]. RCT involves the membrane proteins ATP-binding cassette A1 (ABC-A1), ATP-binding cassette G1 (ABC-G1), and scavenger receptor BI (SR-BI) that participate in cholesterol transport [2, 3]. Nascent HDL resulting from these interactions undergoes further maturation in the plasma via interaction with lecithin cholesterol-acyl transferase (LCAT) to produce mature spherical HDL [4]. Independently of RCT, HDL also mediates anti-inflammatory and anti-oxidant processes [5, 6].

Numerous naturally occurring apoA-I mutants have been identified [7, 8], with the most common functional outcome being impaired interaction with LCAT or an increased propensity to form amyloids [7]. Haase et al. have recently described the previously unknown A164S variant of apoA-I found at a frequency of 1:500 in the Danish general population (10 440 individuals from the Copenhagen City Heart Study, CCHS) [9]. This is the first known variant of apoA-I in which heterozygous carriers have an increased risk of ischemic heart disease (IHD), myocardial infarction (MI) and mortality whilst showing no difference in HDL cholesterol levels, plasma lipids or apoA-I concentration compared to noncarriers [9]. The authors show that HDL maturation in mice expressing apoA-I A164S is comparable to those expressing only WT apoA-I, indicating that interaction with LCAT is unlikely to be a cause of the A164S phenotype [9].

Given the health impact of apoA-I A164S and its high occurrence in the general population (Danish), elucidating the impact of the single amino-acid substitution on protein structure and function is of particular interest. The present study therefore set out to analyze the structure and functional characteristics of apoA-I A164S compared to apoA-I WT and other well-studied variants. As the single point mutation of A164S is localized close to a region of apoA-I (amino acids 173 to 178) in which other mutations result in known variants that exhibit hereditary amyloidosis [10] particular focus was given to investigating the amyloidogenic propensity of the A164S variant in addition to measuring its stability and lipid binding properties.

## Material and Methods

### Production of recombinant protein

A bacterial expression system consisting of pNFXex plasmid in *Escherichia coli* strain BL21(DE3) pLysS cells (Invitrogen) was used to produce the mature forms (243 amino acids) of apoA-I WT and apoAI-A164S proteins, as previously described [10, 11]. Briefly, the apoA-I gene was cloned into the pEXP-5 plasmid (Novagen, Inc, Madison, WI, USA). Primer-directed PCR mutagenesis was used to create the A164S, L178H and G26R mutations. The mutations were verified by dideoxy automated fluorescent sequencing (GATC Biotech). The plasmids were transferred into the bacteria and cultivated at 37°C in LB medium with 50 μg/ml of ampicillin and 34 μg/ml of chloramphenicol. Protein expression was induced for 3–4 h following the addition of 0.5 mM isopropyl-beta-thiogalactopyranoside (Sigma, St Louis, MO, USA). Following cell disruption, apoA-I was purified from the soluble fraction of the cells using a His-Trap-Nickel-chelating column (GE Healthcare, Uppsala, Sweden) and a mobile phase of phosphate-buffered saline (PBS), pH 7.4 with 3 M guanidine. The protein was extensively washed in PBS (pH 7.4) containing 40 mM imidazole, and then eluted with PBS containing 500 mM imidazole. Imidazole was removed from the protein sample by using desalting columns (GE Healthcare, Uppsala, Sweden) equilibrated with PBS, pH 7.4. After purification of apoA-I proteins on Ni^2+^-chelated columns (GE Healthcare) and desalting to remove imidazole, TEV protease treatment was employed to cleave the His-tag. This was followed by a second Ni^2+^- column passage where TEV protease and the cleaved His-tag were retained on the column. The flow-through containing cleaved apoA-I proteins was desalted into phosphate buffered saline, pH 7.4, 150 mM NaCl, concentrated with 10 kDa molecular weight cut-off Amicon Ultra centrifugal filter devices (Millipore) and stored at 4°C prior to use. To confirm the mutation, wild type and mutated proteins were in-solution digested using trypsin. The peptide samples were separated by reversed phase nano-LC using a nanoEasy spray ion source (Proxeon Biosystems, Odense, Denmark) and introduced directly into an LTQ-Orbitrap Velos Pro mass spectrometer (Thermo Fisher Scientific, Bremen, Germany). A database including the sequence for apoA-I protein, both as wild type and the mutated version, was generated and used in the Mascot Server software (v.2.5, http://www.matrixscience.com) to confirm the mutation. Protein purity was confirmed by sodium dodecyl sulfate (SDS)-polyacrylamide gel electrophoresis with Coomassie blue staining and protein concentrations determined by Nanodrop, using molecular weight and extinction coefficients.

### Circular dichroism spectroscopy

Circular dichroism spectroscopy (CD) measurements were performed on a Jasco J-810 spectropolarimeter equipped with a Jasco CDF-426S Peltier set to 25°C. Averages of five scans were baseline-subtracted (PBS buffer; 25 mM phosphate, 150 mM NaCl) and the alpha-helical content was calculated from the molar ellipticity at 222 nm as previously described [10, 12].

For thermal stability experiments spectra were obtained from 25°C to 80°C with 2.5°C increments. ApoA-I was diluted to 0.2 mg/ml in PBS (final concentration was 25 mM phosphate, 150 mM NaCl, pH 7.4), placed in a 0.1 mm quartz cuvette and, after extensive purging with nitrogen, scanned in the region 200 to 260 nm (scan speed was 20 nm/min). The Boltzmann function within the GraphPad software (GraphPad Software, Inc., CA, USA) was used to fit the molar ellipticity values at 222 nm of the temperature gradient to a sigmoidal fit curve.

A stopped flow instrument coupled to CD studied the stability of apoA-I proteins in the presence of SDS. Molar ellipticity (222nm) of apoA-I (0.5 mg/ml) was measured after mixing with 100 mmol/l solution of SDS at a volume ratio 1:5. The decrease of protein signal in a sigmoidal shape was recorded and t1/2 values were determined from Boltzmann fit.

### Thioflavin T (ThT) binding assay

A164S and WT (0.2 mg/ml) were incubated at 37°C and diluted with ThT stock at time of use. 180 μl of protein was incubated for 10 min in the dark with 20 μl of ThT with the final concentration 10 μmol/l (ThT stock: 1 mmol/l stored in the dark at 4°C; Glycine buffer stock: 0.1 mol/l at pH 8.5 stored at 4°C) ThT fluorescence was then measured using a VICTOR3 Multilabel Plate Counter (PerkinElmer, Waltham, MA, USA) spectrofluorometer at an excitation wavelength of 450 nm and an emission wavelength of 545 nm, with excitation and emission slit widths of 10 nm [10].

### Transmission Electron microscopy (TEM)–Negative stain

Protein samples incubated at 37°C for 28 days were analyzed by negative stain electron microscopy. 5μl of apoA-I proteins were adsorbed onto carbon-coated grids for 60 s, and stained with 7 μl of 2% uranyl acetate for 20 s. The grids were rendered hydrophilic by glow discharge at low pressure in air. Specimens were observed in a FEI Tecnai Spirit BioTWIN electron microscope operated at 100 kV accelerating voltage, and images were recorded with a Veleta TEM CCD camera (Lund University Bioimaging Center) [10]. Stability and purity of apoA-I WT, A164S, L178H and G26R in PBS buffer from 0 and 28 days of incubation were detected by SDS PAGE gel analysis.

### Limited proteolysis

Protein (5 μg per reaction) in phosphate buffered saline (pH 7.4, 150 mmol/l NaCl) was treated with 1:2000 wt/wt ratio of high purity chymotrypsin (Sigma-Aldrich) for the indicated periods of times. Reactions were stopped with protease inhibitor cocktail (Roche) followed by addition of SDS loading buffer. Samples were stored at -20°C until analysis with SDS-PAGE.

### Formation of recombinant HDL (rHDL)

Lyophilized DMPC (1,2-dimyristoyl-sn-glycero-3-phosphocholine; Avanti Polar Lipids) was dissolved in 3:1 chloroform:methanol by vortexing and the solvent was completely evaporated by overnight incubation under a stream of nitrogen gas. The phospholipids were then dissolved in PBS by vortexing and the lipid suspension was extruded through a 100 nm polycarbonate membrane using the LiposoFast system (Avestin) to create unilamellar vesicles. ApoA-I WT and apoA-I A164S were incubated at 37°C with unilamellar DMPC at a molar ratio of protein to phospholipid of 1:100 and a protein concentration of 0.4 mg/ml. Samples were taken at 0, 1, 3, 6, 9, 24 h and at 4 days and stored at -80°C with 10% w/v sucrose until analysis by BN-PAGE using the NativePAGE Bis-Tris Gels System 4–16% (Invitrogen) according to the manufacturer’s instructions. The lipid binding capacity was evaluated by a spectrophotometric lipid clearance assay (Thermo Scientific-Multiskan Go, Finland) as described previously [10]. Briefly, unilamellar DMPC vesicles were prepared as described above and placed in a 96-well plate, where lipid-free apoA-I proteins were added at a 1:100 protein to lipid ratio (mol/mol). The spectral changes (at 325 nm) were monitored over time as phospholipid molecules were used in the formation of smaller protein-lipid complexes, with a consequent clearance of larger DMPC vesicles. Although the lipid binding of apolipoproteins is known to be a complex process involving several intermediate steps, this assay is accepted in the field as an approximate measure of the lipid-binding capability of an apolipoprotein.

### ApoA-I ex vivo analysis

Male Sprague Dawley rats purchased from Taconic (Ry, Denmark) were used at the age of 10–11 weeks. Rats were on normal diets. Blood samples were collected and serum samples (200 μl) were mixed with apoA-I WT and A164S (0.75mg/ml). The mixtures were incubated for 2, 4 and 8 h at 37°C. The samples were separated by NativePAGE Bis-Tris Gels System 4–16% (Invitrogen), and transferred to PVDF membranes, probed with anti-human apoA-I antibodies (Abcam) and immune detection performed with HRP-conjugated secondary antibodies (GE Healthcare). Blots were imaged using the Odyssey Fc system (LI-COR) and quantified using Image studio v2.0 software. The animal procedures were approved by the *Malmö/Lund Committee for Animal Experiment Ethics*.

### Cholesterol efflux

The method used for the measurement of cholesterol efflux was adapted from [13, 14]. J774 macrophages (ATTC) were plated into 48-well cell culture dishes at 75,000 cells/well (500 μl/well) in RPMI 1640 (Thermo scientific) supplemented with 10% FBS and gentamicin and after 24 h the media was replaced with 250 μl/well RPMI 1640 containing 5% FBS, 4 μCi/ml ^3^H-cholesterol (Perkin Elmer), 2 μg/ml ACAT inhibitor (Sandoz 58–035, Sigma) and gentamicin. After a further 24 h the ^3^H-cholesterol media was replaced with 500 μl RPMI 1640 supplemented with 0.2% BSA (low free fatty acids and low endotoxin, Sigma), 2 μg/ml ACAT inhibitor, 0.3 mmol/l Cpt-cAMP (Abcam) and gentamicin for 18 h. The cells were washed twice with MEM-HEPES and then triplicate wells were treated with 300 μl/well apoA-I WT or A164S in MEM-HEPES at the indicated concentrations and for relevant durations. Cholesterol efflux was measured by collecting the media, centrifuging at 14,000xg for 5 min at room temperature to pellet any collected cells, and 100 μl supernatant transferred to a scintillation vial. 5 ml of scintillation fluid was added to each sample before scintillation counting was performed (Perkin Elmer). To obtain a measure of ^3^H-cholesterol in the cells before efflux incubations began a set of wells from each experiment were incubated with 1% sodium deoxycholate and the lysate collected for scintillation counting. The mean reading from these samples was considered to be representative of the total cellular pool of ^3^H-cholesterol available for efflux to apoA-I WT or A164S and therefore total efflux for each treatment was calculated as a % of this value. Non-specific background efflux was measured in triplicate for all relevant time points in each experiment and subtracted from time-matched treatments.

### Statistical analysis

To calculate the differences between apoA-I WT and A164S in unfolding experiments using SDS and the lipid clearance assay, a two-tailed unpaired t-test, Boltzmann fit and t1/2 of a one-way decay of non-linear regression were performed. Time-dependent differences in ThT binding of apoA-I WT and G26R were assessed using a two-way ANOVA with Sidak’s multiple comparison test. All data were analyzed using Graphpad Prism (GraphPad Software, Inc., CA, USA). A p value of ≤0.05 was considered significant.

## Results

### Pro-amyloidogenic properties of apoA-I WT and A164S

**Fig 1:**
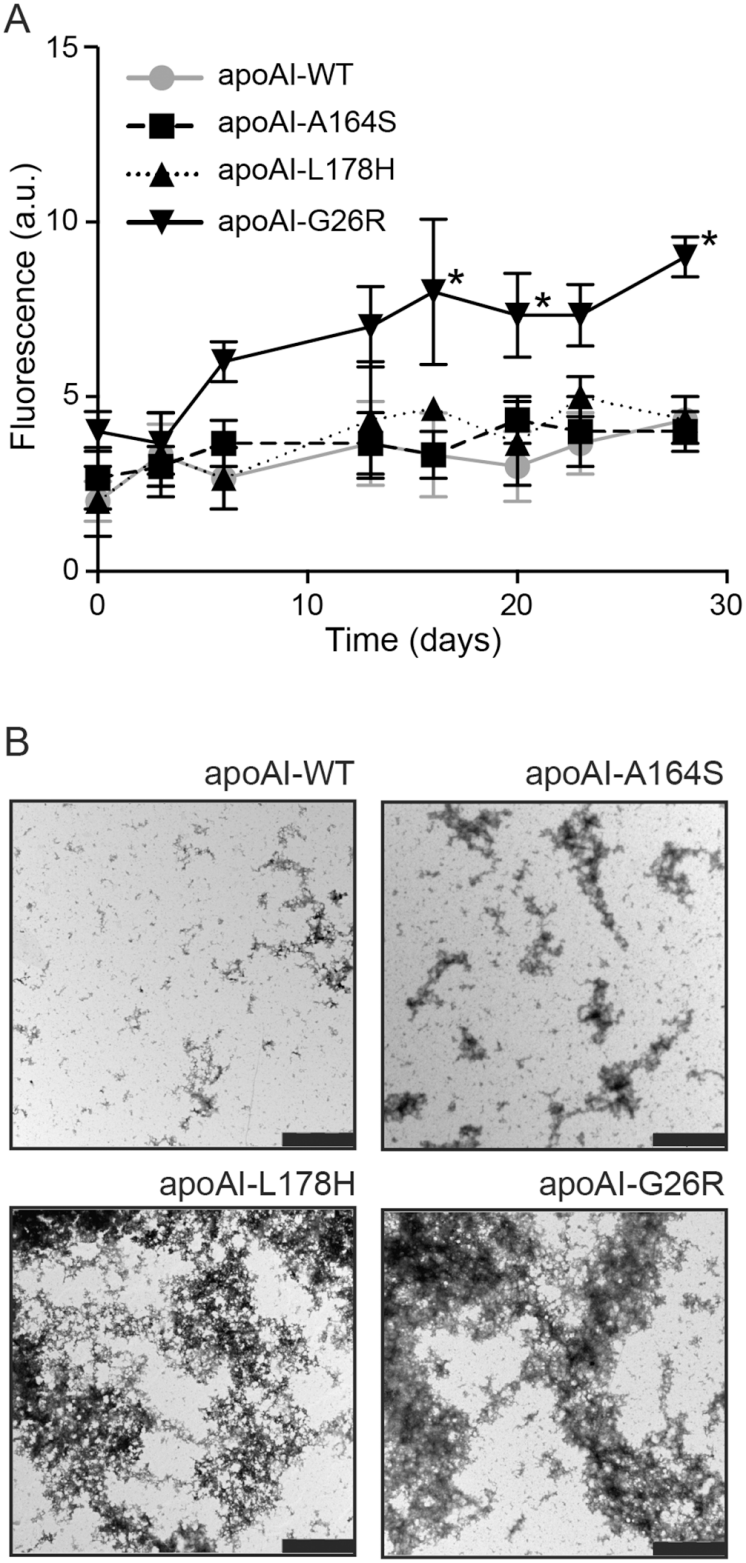
Evaluation of pro-amyloidogenic properties of apoA-I A164S. **A)** Binding of apoA-I WT and variants to the amyloidophilic dye Thioflavin T (ThT). ApoA-I WT, A164S, L178H and G26R were incubated with ThT solution at 37°C and fluorescence measured on day 0, 3, 7, 12, 16, 20, 23 and 28. * p≤0.05 ApoA-I WT vs. G26R matched time point readings, data is mean ±SEM (n = 3). **B)** ApoA-I A164S, apoA-I WT, apoA-I G26R and apoA-I L178H proteins were incubated at 37°C for four weeks followed by negative stain TEM analysis. While the positive control apoA-I G26R and L178H variants formed pre-fibrillar structures and aggregates (bottom panels), apoA-I WT (upper right panel) showed no aggregation and apoA-I A164S (upper left panel) displayed short amorphous aggregation. Bar corresponds to 2 μm.

All variants of apoA-I were stable, with minor visible fragmentation and SDS-resistant oligomer formation after 28 days of incubation at 37°C (not shown). The length of incubation clearly exceeds the half time of apoA-I in circulation (t1/2 for apoA-I is <5 days), however, amyloidogenic apoA-I variants accumulate in tissue and tissue-residing apoA-I may thus have a significantly longer time before being cleared. An extended time of incubation was therefore chosen to take into account this possibility. The ThT assay and EM analysis were used to assess the pro-amyloidogenic propensity of the A164S variant. No difference was observed in amyloidophilic Thioflavin T (ThT) affinity between A164S and WT apoA-I (Fig 1) indicating negligible transition to beta-sheet structure in the A164S variant during the extended 28 days of incubation. We have previously reported high alpha-helical and low beta-sheet content of fibrillar L178H variant which was here employed as a negative control [10] and the G26R variant with strong affinity to ThT dye was employed as a positive control (p≤0.05 vs. apoA-I WT after 16, 20 and 28 days of incubation; Fig 1A) [15]. Negative stain TEM analysis was next used to visualize potential protein assemblies (Fig 1B). ApoA-I WT showed formation of high molecular weight assemblies (0.2–1 μm diameter) but fewer and less elongated fibers compared to all other variants analyzed. Similarly, the EM images revealed elongated aggregates of the A164S variant after 28 days of incubation at 37°C but to a much lesser extent than apoA-I L178H and G26R, which exhibit significantly greater aggregation under the same experimental conditions.

### Secondary structure and stability of apoA-I A164S

**Fig 2:**
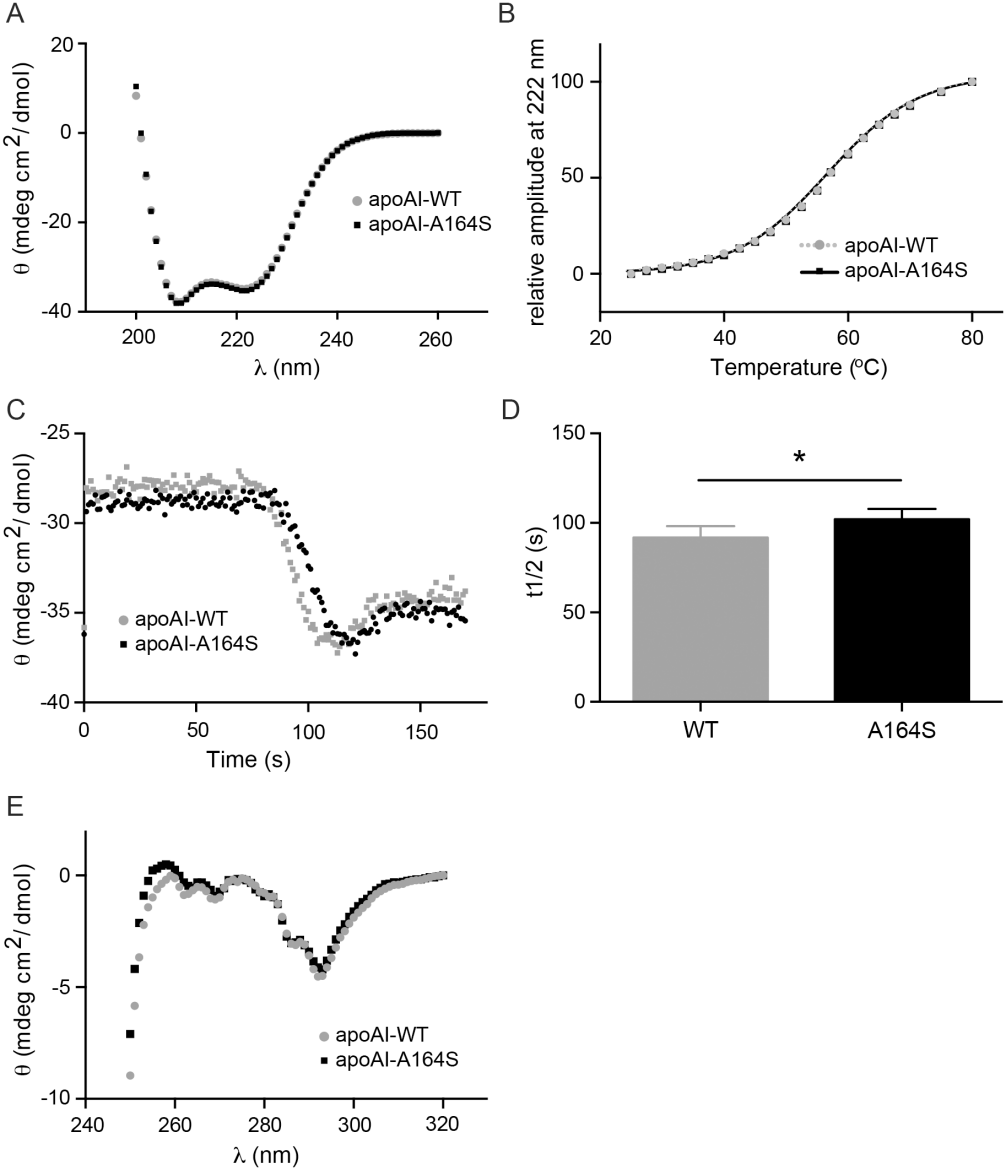
Analysis of alpha-helical content and thermal stability of apoA-I A164S. (A) CD analysis of apoA-I WT and A164S. Alpha-helical content of 0.2 mg/ml of apoA-I was calculated from molar ellipticity at 222 nm (n = 3). (B) Thermal stability of 0.2 mg/ml of apoA-I was assessed from normalized sigmoidal decrease of molar ellipticity at 222 nm. The results are mean ± SEM (n = 3). (C) Stopped-flow coupled to CD: apoA-I WT and A164S (0.5 mg/ml final concentration) were mixed with 100 mM SDS at the volume ratio 1:5 and molar ellipticity at 222 nm was measured. (D). Bar graph shows the t1/2 of apoA-I WT and A164S. Data is mean ±SEM (* = p<0.05, n = 6). (E) Near CD analysis of apoA-I WT and A164S (1.25 mg/ml).

Far UV CD spectra suggest a similarity in secondary structure between apoA-I WT and the A164S variant. Alpha-helical content, calculated from 222 nm molar ellipticity, showed no significant difference between apoA-I WT and apoA-I A164S (60±0.8% and 59±0.4% helical content, respectively) (Fig 2A). This degree of alpha-helical content of apoA-I WT is consistent with our previous work and that reported by others [16, 17]. Melting points of apoA-I WT and A164S were also measured and suggest almost identical thermal stability (57.3±0.4°C and 57.2±0.2°C, respectively, Fig 2B).

The stability and unfolding characteristics of apoA-I proteins in the presence of anionic surfactant were studied by a stopped flow instrument coupled to CD. Molar ellipticity (222nm) of apoA-I (0.5 mg/ml) was measured after mixing with 100 mM solution of SDS at a volume ratio 1:5 (Fig 2C). The decrease of protein signal in a sigmoidal shape was recorded and t1/2 values were determined for WT and A164S to 92±9s and 102±8s, respectively (p = 0.043) (Fig 2D).

### Tertiary structure of apoA-I A164S

**Fig 3:**
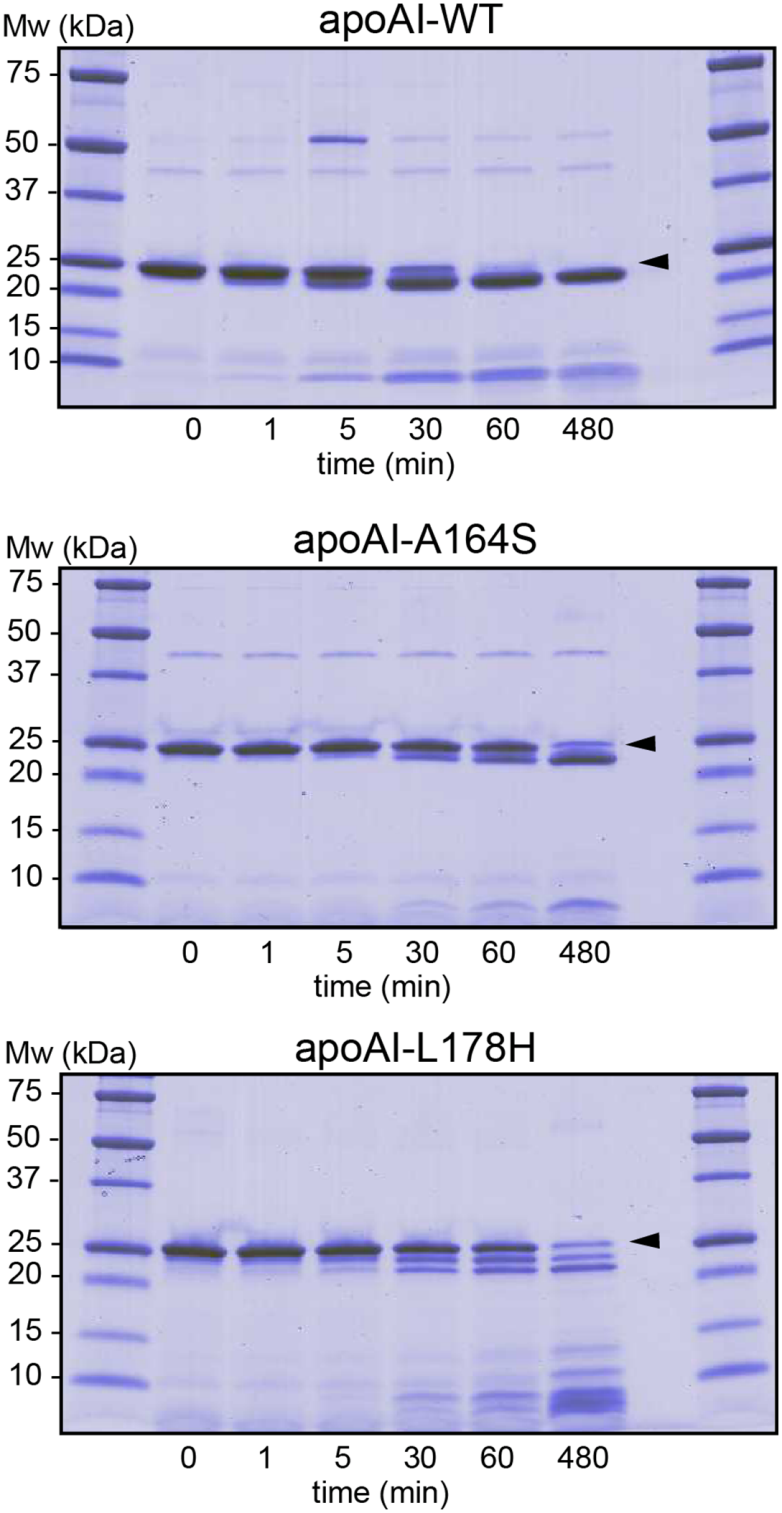
Limited proteolysis analysis. ApoA-I WT, A164S and L178H (5μg) were incubated for indicated times at 37°C in the presence of chymotrypsin. The cleaved products from limited proteolysis from different time points were separated by SDS-PAGE and visualized by coomassie staining. *Arrows* indicate migration distances of full-length proteins.

Near CD analysis of the tertiary structure of apoA-I WT and A164S (1.25 mg/ml) showed that both proteins have similar folding properties (Fig 2E). The signals of aromatic amino acid side chains, in the region from 320 nm to 250 nm, suggest that the mutation resulting in the replacement of an alanine with serine at position 164 of apoA-I does not cause significant changes in the tertiary structure compared to WT protein. The results are in good agreement with limited proteolysis experiments (Fig 3) showing similar profiles of cleaved fragments between WT and A164S indicating that these proteins are almost identically folded and that chymotrypsin cleaves short fragments from the C-terminal domain of the protein as we have previously reported [10, 15]. The L178H variant exhibits loose tertiary structure with a less structured N-terminal domain resulting in a distribution of cleavage fragments from the N-terminus as shown in Fig 3, which are different from those observed for WT and A164S, and in agreement with a previous report on the L178H variant [10].

### The A164S point mutation affects functional properties of apoA-I

**Fig 4:**
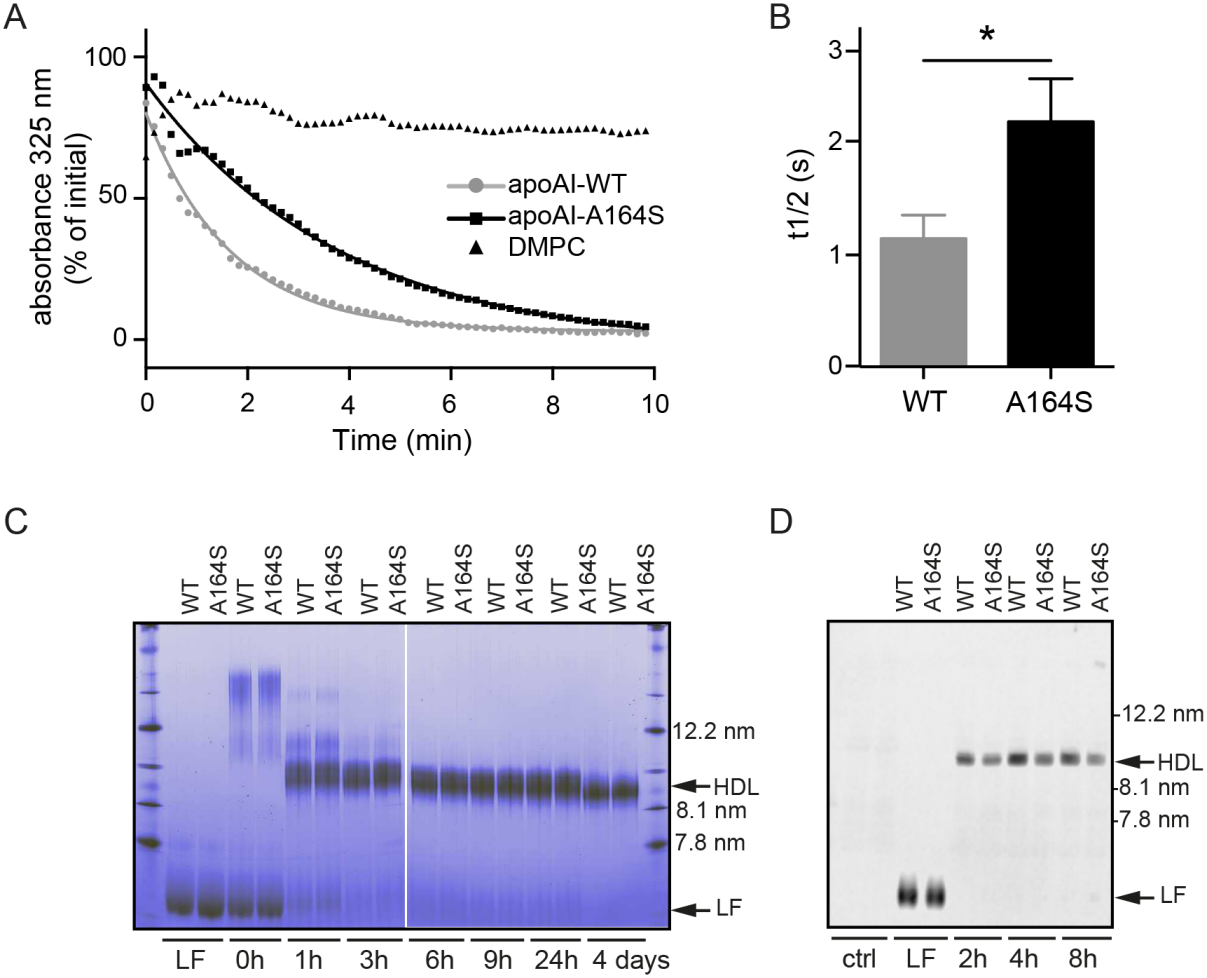
Lipid clearance assay and native gel analyses of rHDL formation. (A) ApoA-I WT and A164S were combined with DMPC at a 1:100 molar ratio and lipid binding measured by absorbance at 325nm at indicate times. Readings were fitted to one-way decay of non-linear regression. (B) Bar graph shows the lipid clearance rate described as t1/2 values calculated from panel A. Data are mean ±SEM (* = p<0.05, n = 3). (C) ApoAI-A164S and apoA-I WT were incubated with DMPC lipids at 37°C for indicated times and analyzed by native gel. *Arrows* indicate apoA-I WT and apoA-I A164S rHDL particles of diameters of approximately 10 nm. Lipid-free (LF) proteins at time 0 h prior to mixing with DMPC lipids are included as controls. (D) Formation of HDL upon incubation of apoA-I WT and A164S with rat serum. 0.75mg/ml of WT or A164S apoA-I was incubated with 200 μl rat serum. Samples were collected at indicated times and equal amounts of protein (0.56μg) separated by blue native PAGE and western blotting for human apoA-I was performed. Lipid-free (LF) proteins (not incubated with serum) and serum without protein (ctrl) are included as controls.

As the main action of apoA-I is in the binding of cellular phospholipids and cholesterol we applied quantitative and qualitative lipid assays to determine the functional properties of the A164S variant (Fig 4). Using a short-term lipid clearance assay we show that the rate of phospholipid binding, expressed as t1/2 calculated from fitting to a one-way decay of non-linear regression, is significantly greater for apoA-I A164S (2.2±0.2 min) compared to apoA-I WT (1.1±0.1 min; p = 0.014, Fig 4A and 4B). After 10 min of incubation with lipids both WT and A164S variant reached the same equilibrium. Changes in protein oligomerization were used as a qualitative assessment of lipid clearance over a longer duration than the short-term lipid clearance assay. Incubations with lipid over hours to days resulted in similar oligomer profiles and sizes of rHDL particles produced by WT and A164S (Fig 4C). The diameter of rHDL particles with WT and A164S after 4 days of incubation was approximately 10 nm. To validate this observation under more physiological conditions, apoA-I WT and A164S were combined with rat serum and oligomerization was monitored at various time points by western blotting of membranes derived from native PAGE with an apoA-I antibody recognizing only the human protein. In agreement with the lipid clearance assay, both WT and A164S produced the same size of HDL (Fig 4D).

### Cholesterol efflux

**Fig 5:**
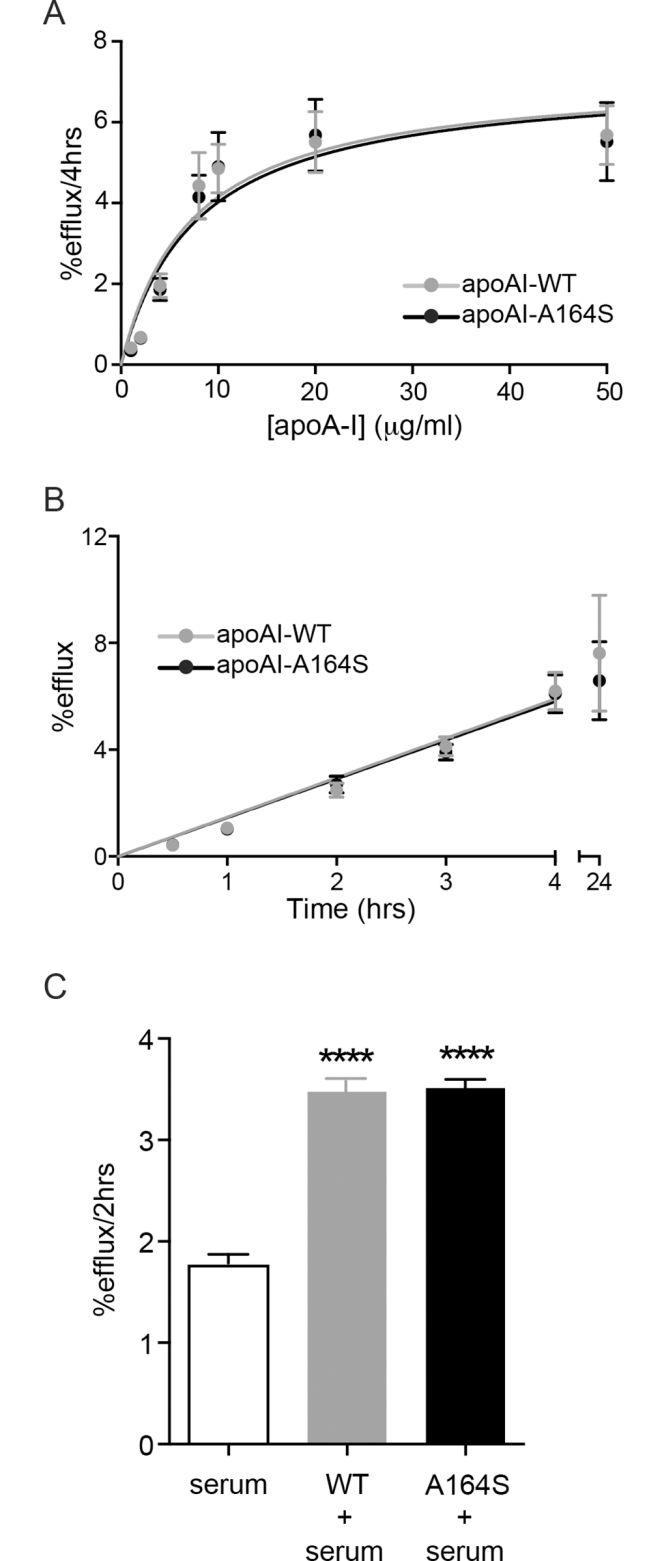
Cholesterol efflux from macrophages. J774 macrophages enriched with ^3^H-cholesterol were incubated with apoA-I WT or A164S and cholesterol efflux quantified as a function of protein concentration (A) or time (B) by scintillation counting of the resulting treatment media. An ACAT inhibitor and CPT-cAMP were used to prevent formation of cholesteryl esters of the ^3^H-cholesterol and to induce expression of ABCA1, respectively. Data from concentration gradient experiments were fitted using the Michaelis-Menten equation. Time dependent efflux treatments were performed with 50 μg/ml apoA-I WT or A164S. Each figure represents 3 independent experiments and displays mean±SD. (C) ApoA-I WT or A164S were incubated with rat serum for 2 h followed by incubation with J774 macrophages enriched with ^3^H-cholesterol for 2 h (10 μg/ml apoA-I). Rat serum was used as control for background efflux. Data is mean ±SEM (**** = p<0.0001, n = 3).

To compare the functionality of apoA-I WT and A164S in a more physiological system cholesterol efflux from macrophages was employed. As described in the Material and Methods section, the cells where loaded with radioactive cholesterol, and preincubated with the cAMP analogue, Cpt-cAMP, to induce expression of the ABCA-1, followed by incubation with the apoA-I proteins. Fig 5A and 5B shows that the ability of the lipid-free A164S variant to efflux cholesterol did not differ from lipid-free apoA-I WT in neither the concentration-dependent (WT apoA-I Vmax = 7.17±0.64%efflux/4h, Km = 7.30±1.83μg/ml, A164S Vmax = 7.14±0.70%efflux/4h, Km = 7.76±2.1μg/ml) nor the time-dependent (apoA-I WT 1.45±0.089%efflux/hr, A164S 1.48±0.089%efflux/hr) experimental analyses. The standard efflux protocol used in Fig 5A and 5B uses lipid-free apoA-I protein which provides useful information about apoA-I function, however under physiological conditions the majority of circulating apoA-I will be bound to lipids in various species of HDL. Based on the lipid clearance assay findings (Fig 4A and 4B) we hypothesized that a difference in lipid association may occur between WT and A164S apoA-I in serum which could influence efflux capacity. By incubating apoA-I WT and apoA-I A164S in rat serum for 2 h native-like HDL particles were formed (Fig 4D) and then used to test if cholesterol efflux from cholesterol-loaded J774 macrophages was altered after binding with physiologically relevant lipids (as shown in Fig 4D). Both apoA-I WT (3.48±0.13%efflux/2h) and A164S (3.51±0.11%efflux/2h) showed significantly higher efflux capacity compared to serum only (1.76±0.11%efflux/2h, p = ≤0.0001) (Fig 5C). However, there was no difference between the two proteins. HDL formed from longer incubations of apoA-I WT and A164S with serum (4h and 8h) gave the same result as that shown in Fig 5C (not shown).

## Discussion

The failure of many pharmacological trials, and a recent genetics study, to directly relate increased HDL-cholesterol (HDL-C) to reduced cardiovascular events [18, 19] indicates that the mechanism by which HDL functions is more complex than the epidemiological evidence suggests [20, 21]. Khera et al have shown that HDL cholesterol efflux capacity is inversely associated with coronary artery disease, independently of HDL-C, highlighting the importance of HDL function rather than concentration to its associated cardio-protective effects [22]. This observation is exemplified by the seemingly paradoxical low frequency of atherosclerosis in heterozygous carriers of apoA-I Milano (A173C) despite low plasma levels of HDL-C and apoA-I, and hypertriglyceridemia [23, 24]. In contrast, apoA-I A164S carriers have normal HDL-C and apoA-I plasma concentrations but increased incidence of IHD and MI [9], and so similarly to apoA-I Milano demonstrates a fundamental disconnection between HDL-C concentration and heart disease. Similarly, the HDL research field is likely to benefit from an improved understanding of A164S so we have therefore conducted the first structure and functional analysis of this protein.

**Fig 6:**
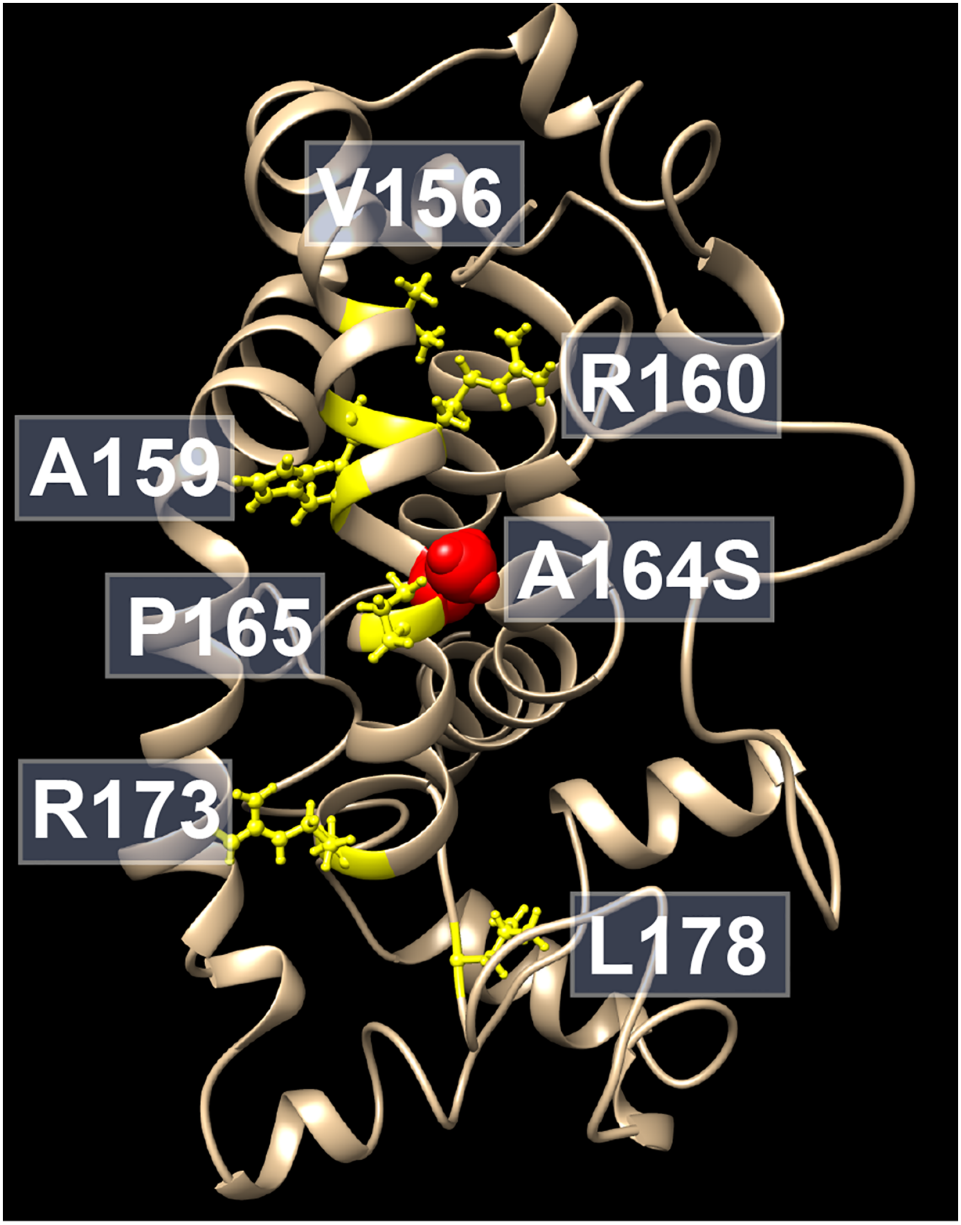
ApoA-I structure model. Location of mutations (*yellow*) in the vicinity of the A164S variant (*red*) that are linked to hereditary amyloidosis, or associated with LCAT deficiency and low HDL levels, are depicted in the lipid-free structure model [52].

Several mutations in apoAI proteins are associated with deficiency in formation of HDL, as well as in different forms of dyslipidemia and hereditary amyloidosis. Two single point mutations of apoAI (R173P and L178H) are those known to be in close proximity of to the A164S variant and are linked to hereditary amyloidosis [25, 26] (Fig 6). Moreover, numbers of point mutations in the apoA-I protein (R160V; H162A; V156E; A159R; L159P; R160L; P165R and R173C), which are in the same region as the A164S variant, are associated with LCAT deficiency and low HDL levels in both animal models and humans [7, 27, 28]. However, some of mutations of apoA-I seem not to affect plasma levels of HDL (H162Q; E169Q and R177H) [7, 28–30]. This indicates that the tertiary structure and correct protein folding are necessary components for fully functional apoA-I proteins.

Our biophysical measurements of the secondary and tertiary structure, and stability of the lipid free apoA-I A164S variant show overall similarity to apoA-I WT. One of the main outcomes of deletion and non-synonymous apoA-I mutations is the formation and pathological deposition in various tissues of amyloid deposits [31, 32]. Although no evidence of aggregates were found in abdominal fat biopsies from A164S carriers [9], amyloidogenic apoA-I proteins are known to affect a number of tissues and confirmation by direct analysis of the A164S protein is therefore required, which we undertook here. We have previously characterized the amyloidogenic apoA-I L178H as forming helical fibrils, which is in contrast to the increased beta-sheet content of the apoA-I G26R variant during amyloidogenesis [10, 15]. Using these variants as controls for alternative amyloid structures, the ThT binding assay, which detects beta-amyloids, showed no difference between apoA-I A164S, WT or L178H. However, EM analysis, to monitor overall aggregation, showed observably more very short (relative to the amyloidogenic controls) elongated aggregates for apoA-I A164S compared to WT, but far less aggregation than that shown for L178H and G26R. Based on the fat biopsy data [9] and our in vitro observations we propose that the A164S variant does not have strong amyloidogenic propensity. However, as in vivo investigations extended to other tissues have not been conducted we cannot exclude the possibility that this weak propensity to aggregate is physiologically relevant. Protein aggregates are known to accumulate in specific tissues [33] and it remains possible that apoA-I A164S can preferentially aggregate in the atherosclerotic vascular wall where the acidic microenvironment may facilitate amyloid formation [34]. Although there are practical difficulties, analysis of relevant cardiac tissue biopsies from A164S carriers as well as analysis for amyloid deposits in animals treated with the variant would help to clarify this matter.

The principal function of apoA-I is to transport cholesterol from peripheral tissues for delivery to the liver for reprocessing or secretion. The ability of this protein to interact and associate with lipids and bind receptors mediating lipid transfer is vital to its function so we therefore assessed these characteristics of the A164S variant. ApoA-I interacts with anionic surfactants to attain a highly helical lipid-like secondary structure. Electrostatic and hydrophobic interactions between positively charged apoA-I and negatively charged surfactant head groups help micelles to bind to the surface of proteins [35]. A substitution of the polar amino acid serine in place of the hydrophobic amino acid alanine at position 164 therefore provides a possible explanation to the faster binding of SDS to WT compared to A164S. This notion is supported by the orientation of the side-chain at position 164, which is located to alpha-helical structure in both the lipid-free [36, 37] and lipid bound [38–40] states and according to the crystal structure of apoA-IΔ(185–243) (this is a lipid-free structure but more likely resembles the extended circular structure of lipid-bound apoA-I in HDL) points to the lipid acyl-chain in the interior of the phospholipid bilayer [39]. The same principal is involved in the interaction of apoA-I with phospholipids, which is a probable explanation for the significantly slower rate of lipid clearance by A164S compared to WT in short-term experiments (10 min). Steric hindrance caused by close proximity of the more bulky serine (as compared to alanine) to the neighboring proline residue (P165) may also contribute to the impaired initial lipid-binding of the apoA-I A164S variant. From our earlier analyses on apoA-I WT structure we know that the region around residue 164 is part of the interface between apoA-I polypeptides in oligomeric organizations of the lipid-free protein [41, 42]. Substitution to a more polar residue may therefore contribute to the stability of oligomeric apoA-I, which in turn may affect initial lipid-binding. As lipid-free and lipid-poor apoA-I are known to be preferentially cleared from the circulation by the kidney [43] a possible consequence of the impaired short-term lipid binding of A164S is a more rapid clearance of newly synthesized or delipidated A164S from the blood. Although this hypothesis has yet to be analyzed it is supported by the finding that there is a mean 13% reduction in A164S plasma concentrations compared to WT apoA-I in A164S heterozygotes [9], which the authors postulate may be a result of enhanced catabolism. However, given that total apoA-I plasma concentrations in A164S carriers are equivalent to noncarriers, increased A164S catabolism due to impaired short-term lipid binding is unlikely to explain the disparity in cardiovascular disease and mortality between these populations. Although the relevance of this result cannot be discounted, longer term treatments (2–8 h) to assess HDL formation in rat serum and the cholesterol efflux assay, both with lipid-free apoA-I protein and lipid-bound showed no difference in function between the A164S variant and WT apoA-I. This demonstrates that the lipid-free A164S protein appears to function normally, even though the initial phase of lipid binding in solution is impaired.

While our work provides important information about the structure and functional characteristics of apoA-I A164S in the apo state, it is currently unknown how this mutation influences HDL maturation and lipid composition. The maturation of HDL is complex and involves multiple stages of constituent lipid modification and flux between other lipoproteins, mediated by several different enzymes, as well as related changes in the spatial arrangement and oligomeric association with apoA-I and other apolipoproteins [43]. It has been reported that not only do total levels, size, and composition of HDL play important role in cardiovascular protection but that the different subclasses of HDL have varying efficacies [44, 45]. It has been shown that apoA-I-deficient mice expressing either WT alone or both WT and A164S apoA-I have comparable plasma profiles of HDL, suggesting that the interaction with LCAT and subsequent remodeling is unaffected by this amino acid substitution [9]. Despite this qualitative similarity, there are now well recognized pleiotropic actions of apoA-I/HDL that are relevant to cardiovascular disease, including anti-inflammatory [46] and antioxidant effects [47] (both referred to by [9]), anti-apoptosis [48] and an involvement in glucose uptake and lipid metabolism [12, 49–51]. Dysfunction in the ability of apoA-I A164S to mediate any of these effects may link this variant to increased cardiovascular disease. Similarly, comparative mass-spectroscopy studies on apoA-I A164S and apoA-I WT from human plasma samples to monitor post-translation modification patterns (glycosylations, oxidations, glycations etc.) may reveal qualitative and functional differences that we cannot find in apoA-I protein purified from a prokaryotic host organism. The use of such techniques to compare WT and A164S HDLs could reveal interesting information regarding important determinants of HDL function.

Overall, our biophysical and biochemical comparisons of the structure and functional properties of the newly discovered apoA-I A164S shows no clear differences to apoA-I WT in stability, secondary structure, size of HDL formed from serum incubation nor cholesterol efflux capacity. It remains possible that either the minor tendency for aggregation or the impaired short-term lipid binding of apoA-I A164S are the source of the associated cardiovascular pathology, however more focused studies on these specific results along with analysis on the lipidome and proteome of A164S HDLs and comparisons of the pleiotropic actions of apoA-I A164S to WT are required.
